# Analysis of the dysregulation between regulatory B and T cells (Breg and Treg) in human immunodeficiency virus (HIV)-infected patients

**DOI:** 10.1371/journal.pone.0213744

**Published:** 2019-03-27

**Authors:** Carolina Gutiérrez, Jacobo Lopez-Abente, Verónica Pérez-Fernández, Adrián Prieto-Sánchez, Rafael Correa-Rocha, Santiago Moreno-Guillen, María-Ángeles Muñoz-Fernández, Marjorie Pion

**Affiliations:** 1 Molecular Immunovirology Laboratory, Department of Infectious Diseases, Ramón y Cajal Health Research Institute (IRYCIS), Ramón y Cajal University Hospital, Madrid, Spain; 2 Immuno-Regulation Laboratory, University General Hospital Gregorio Marañón, Health Research Institute Gregorio Marañón (IiSGM), Medicine and Experimental Surgery Building, Madrid, Spain; 3 Molecular ImmunoBiology Laboratory, University General Hospital Gregorio Marañón, Health Research Institute Gregorio Marañón (IiSGM), Spanish HIV HGM BioBank, Madrid, Spain; 4 Networking Research Center on Bioengineering, Biomaterials and Nanomedicine (CIBER-BBN), Madrid, Spain; Purdue University, UNITED STATES

## Abstract

This study examines the relationship between regulatory B (Breg) and T (Treg) compartments, which play crucial roles in the maintenance of immune homeostasis in the context of HIV. Using flow cytometry, the phenotypes of different Breg and Treg subsets from HIV-infected and healthy individuals were analyzed, along with the suppressive capacity of Breg. Peripheral blood samples of thirteen HIV^+^ treatment-naïve individuals, fourteen treated-HIV^+^ individuals with undetectable viral load and twelve healthy individuals were analyzed. The absolute counts of Breg and Treg subsets were decreased in HIV^+^ treatment-naïve individuals in comparison to treated-HIV^+^ and healthy individuals. Interestingly, correlations between Breg subsets (CD24^hi^CD27^+^ and PD-L1^+^ B cells) and IL-10-producing Breg observed in healthy individuals were lost in HIV^+^ treatment-naïve individuals. However, a correlation between frequencies of CD24^hi^CD38^hi^ or TIM-1^+^-Breg subsets and Treg was observed in HIV^+^ treatment-naïve individuals and not in healthy individuals. Therefore, we hypothesized that various Breg subsets might have different functions during B and T-cell homeostasis during HIV-1 infection. In parallel, stimulated Breg from HIV-infected treatment-naïve individuals presented a decreased ability to suppress CD4^+^ T-cell proliferation in comparison to the stimulated Breg from treated-HIV^+^ or healthy individuals. We demonstrate a dysregulation between Breg and Treg subsets in HIV-infected individuals, which might participate in the hyper-activation and exhaustion of the immune system that occurs in such patients.

## Introduction

HIV infection induces a general dysregulation of the immune system (IS), which can be defined as unrestrained or unregulated immune responses. In the case of the HIV, this implies a general loss of immune cell function and chronic inflammation, which lead to immune exhaustion, where almost all cells of the IS lose their functional ability. B and T cell exhaustion is characterized by an increase of the activated phenotype, a decrease of proliferative ability, and the loss of their effector capacity. These outcomes are related to uncontrolled viral persistence and disease progression [1, 2]. Recently, regulatory B and T cells (Breg and Treg, respectively) have been described to participate in the maintenance of immune homeostasis, of which one aim is to suppress the over-reaction in the case of inflammation, which leads to an appropriate immune response [3–5].

Breg are immunosuppressive cells that support immunological tolerance, and several subsets of Breg have been defined such as CD19^+^CD24^hi^CD38^hi^ [6], CD19^+^CD24^hi^CD27^+^ [7], CD19^+^CD5^+^CD1d^hi^ [8], T-cell immunoglobulin and mucin domain 1 CD19^+^ (TIM-1^+^ B cells) [9], programmed death-ligand 1 CD19^+^ (PD-L1^+^ B cells) [10], CD19^+^CD73^-^CD25^+^CD71^+^ [11], CD19^+^CD39^hi^ [12] or CD19^+^CD23^+^sIgM^hi^sIgD^hi^CD21/CD35^hi^ marginal zone precursor B cells [13]. Currently, the IL-10 expression is the only clear marker defining a suppressive B-cell population in mice and humans, although more recently, Breg function has also been defined as independent of IL-10 [14, 15]. The mechanism of action of Breg in HIV infection is not yet well established, but the frequency of some Breg subsets is dysregulated in the chronic phase of HIV infection and loses its suppressive function over HIV-specific T cells [16, 17].

The loss of the function of HIV-specific CD8^+^ T lymphocytes is also attributed to Treg in HIV infection [16, 18–21]. Treg is a subset of CD4^+^ T cells that control hyper-activation of the IS due to their suppressive capacity [22]. Their role in HIV infection is still debated, and studies on Treg dysregulation and function in HIV^+^ patients have shown dual impacts. Therefore, high levels of Treg in HIV^+^ patients are related to reduced immune activation [23]. However, a high level of Treg is related to a high viremia associated with poor immune restoration [24] and persistence of the HIV reservoir [25]. The relation between Breg and Treg is not well established in HIV infection, but it might be assumed that they are implicated in the general imbalance of the IS since both compartments are dysregulated [20, 26].

We analyzed different Breg subsets and the Treg compartment to understand their interrelation in HIV infection better. We determined that frequencies of Breg and Treg were significantly higher in HIV^+^ treatment-naïve individuals, but their absolute numbers were significantly decreased in the peripheral blood of these individuals. On the other hand, we observed that some Breg subsets are associated with the frequency of IL-10-producing B cells in healthy conditions. However, this correlation was lost in HIV^+^ individuals, which can show impairment in the Breg function during HIV infection. On the other hand, other Breg subsets were related to Treg homeostasis in HIV^+^ treatment-naïve individuals. Therefore, we demonstrated that frequencies and absolute counts of the Breg and Treg subsets, and the suppressive function of the Breg compartment were dysregulated during HIV infection. Understanding the mechanisms and the extent of cellular imbalance is pivotal to determine the development of the disease in HIV^+^ patients and creating more efficient therapies that prevent the premature loss of functional subsets of immune cells.

## Materials and methods

### Patients

All individuals were recruited under a protocol that was approved by the Clinical Ethics Committees of the Ramón y Cajal Hospital and the Gregorio Marañón Hospital and according to the principles of the Declaration of Helsinki (Clinical Ethics Committees references: RyC-419-14 and HGUGM-04/2015, respectively). The data analysis was based on anonymized routine clinical data. Written informed consent was obtained from all the study participants. The research was conducted between December 2014 and December 2016. All samples were freshly processed on the day of the blood extraction within 1 h after sample collection.

We studied three groups of individuals: i) HIV-infected treatment-naïve individuals (Naïve). This group comprised newly diagnosed HIV-1^+^ patients who did not receive antiretroviral treatment and presented a detectable viral load in plasma (VL >50 copies/mL); ii) treated-HIV-1^+^ individuals (Treated); HIV^+^ patients who received antiretroviral treatment and presented an undetectable viral load (VL < 50 copies/ml in plasma), and iii) healthy individuals (Control); individuals who were not infected by HIV and were considered as a control group. A positive diagnostic of HIV-1 was obtained by enhanced ELISA assays that detected both HIV antibody and antigen (4th generation assay) which enables earlier detection of HIV seroconversion, followed by confirmation by Western Blot.

### Direct labeling of whole blood and intracellular labeling

Whole blood was labeled, lysed, and analyzed by flow cytometry. The antibodies and fluorochromes used in this work are shown in the S1 and S2 Tables. The absolute numbers of immune subsets were determined using Flow-Count Fluorospheres and Flow-Count method (Beckman Coulter, Nyon, Switzerland). Peripheral blood mononuclear cells (PBMCs) were isolated with Lymphocyte Isolation Solution (Rafer SL, Zaragoza, Spain). To detect IL-10-producing Breg, 1x10^6^ PBMCs were stimulated with CD40L (Alexis Biochemical; 200ng/ml, Barcelona, Spain), LPS (Sigma-Aldrich; 1μg/ml, Darmstadt, Germany) and CpG-B oligodeoxynucleotide-2006 (CpG; Eurogentec SA; 10μg/ml, Seraing, Belgium) for two days. This mix of *stimuli* was described to induce the Breg phenotype *in vitro* [7]. Further, cells were activated with phorbol 12-myristate 13-acetate (PMA, Sigma-Aldrich; 10ng/ml) and ionomycin (Sigma-Aldrich; 0.25μg/mL) for 5 h at 37°C in 5% CO_2_. GolgiStop (BD Biosciences, Madrid, Spain) was added after the first 1 h of PMA/ionomycin stimulation. The cells were then stained for surface markers, followed by intracellular staining (IL-10) using a Cytofix/Cytoperm kit (BD Biosciences, San Jose, CA, USA). To detect Treg, 1×10^6^ freshly isolated PBMCs were stained for surface markers followed by intracellular staining using a Foxp3/Transcription Factor Staining Buffer Set (eBiosciences, Waltham, Massachusetts, USA). The cells were then analyzed by flow cytometry by using a Gallios cytometer (Beckman Coulter), and data were analyzed using Kaluza Analysis software (Beckman Coulter).

### Definition of clinical markers and cellular phenotypes

The CD4 nadir and the CD4 nadir count are the lowest points to which the CD4 frequency and CD4 count dropped and were obtained from the patients’ clinical records. The CD4/CD8 ratio reflects immune system health and was calculated using the frequencies of CD4 and CD8 quantified at the sampling [27, 28].

We determined the Treg cell subsets in whole blood, which are defined as total Treg (CD127^neg^ CD25^+^) [29], activated Treg (CD127^neg^CD25^+^CD45RO^+^HLA-DR^+^), and terminally differentiated effector cell Treg (TemRA, CD127^neg^CD25^+^CD27^neg^CD45RA^+^), which were all gated on CD4^+^ T cells (S1A Fig). Frequencies of Treg and Treg subsets were determined relative to the CD4+CD127negCD25+ gate. Exhausted CD4^+^ T cells were defined as PD-1^hi^ gated on total CD4^+^ T cells (S1B Fig).

For B-cell subsets, we analyzed the Breg subsets CD24^hi^CD38^hi^, CD24^hi^CD27^+^, TIM-1^+^, and PD-L1^+^, which were all gated on CD19^+^ cells (S2 Fig). The total IL-10-producing stimulated, or unstimulated B cells were determined by the labeling of the isolated B-cell culture as IL-10^+^ in total CD19^+^ cells (S3A and S3B Fig). We also determined the frequencies of IL-10-producing Breg; CD19^+^CD24^hi^CD38^hi^IL-10^+^, and CD19^+^CD24^hi^CD27^+^IL-10^+^ (S3 Fig), frequencies were determined into the CD19^+^CD24^hi^CD38^hi^ and CD19^+^CD24^hi^CD27^+^ gates, respectively (S3C–S3E Fig).

### Proliferation assay

B cells were isolated from PBMCs using anti-human CD19 microbeads (Miltenyi Biotec, Bergisch Gladbach, Germany) with purity > 96%. They were then treated for two days with CpG-B/CD40L/LPS, which is a mixture that induces the Breg regulatory phenotype. Unstimulated B cells are B cells cultured with only the medium and were used as a negative control. After two days, stimulated and unstimulated B cells were washed with PBS (Biochrom, Cambridge, UK) before co-culture. Next, autologous CFSE-labeled CD19-depleted PBMCs (50,000 cells) (CFSE from Life technologies, Alcobendas, Spain) were co-cultured with stimulated or unstimulated-B cells (100,000 cells) and then stimulated with 50,000 anti-CD3/anti-CD28-coated magnetic beads (Dynabeads, Life technologies). After 72 h, the cells from the co-cultures were harvested and labeled with anti-CD4 and anti-CD8 antibodies (Beckman Coulter). The viability was observed using 0.5 μg/ml of 7-amino-actinomycin D (7AAD, Sigma-Aldrich). The frequencies of proliferating CD4 and CD8 T cells were determined by flow cytometry following the loss of CFSE signal to assess the suppressive capacity of B cells. The percentage of suppression of proliferation of CD4^+^ or CD8^+^ T cells was calculated as follows: frequency of suppression of proliferation = 100-(proliferation of CFSE+-labeled cells in co-culture with stimulated B cells*100/ proliferation of CFSE-labeled T cells in co-culture with US-B cells).

### Statistical analysis

A non-parametric Kruskal-Wallis ANOVA test was performed to compare cell populations between healthy individuals (control) and HIV-1^+^ treatment-naïve (Naïve) or treated-HIV-1+ individuals (Treated), as well as to study the suppressive function of B cells. Mann-Whitney tests with Bonferroni correction for multiple testing were then used when the conditions presented significance (p<0.05 in the Kruskal Wallis test). The data are represented as the median ± interquartile range (IQR). The statistical correlation between variables was calculated using a Spearman rank correlation analysis. *P* values below 0.05 were considered statistically significant. All analyses were performed using SPSS 17.0 (IBM, Chicago, USA).

## Results

### Clinical composition

We evaluated 39 individuals who were divided into three groups. There were 13 HIV-infected treatment-naïve individuals with a detectable plasma viral load (VL>50 copies/mL; Naïve), 14 treated-HIV^+^ individuals (Treated), and 12 healthy individuals (Control). The three groups were homogeneous according to age and sex (Table 1).

**Table 1:** Characteristics of HIV^+^-infected and healthy individuals enrolled for the study.

	Control (a)	Naive (b)	Treated (c)	Control vs Naive	Control vs Treated	Treated vs Naive
	Median[Q1-Q3]	Median[Q1-Q3]	Median[Q1-Q3]	p-value	p-value	p-value
n	12	13	14	-	-	-
Gender, male (%)	85	93	75	1,000 ‡	1,000 ‡	1,000 ‡
Age (years)	**39.0** [24.0–46.5]	**36.0** [25.8–50.5]	**41.5** [35.0–53.0]	1,000 ‡	0,755 ‡	1,000 ‡
Years Diagnosed	-	-	**5.0** [3.0–9.5]	-	-	-
Plasma viral load (copies/ml)	-	63096 (1000–1258925)	ND	-	-	-
Years under HAART	-	-	**4.0** [2.7–8.2]	-	-	-
Years with undetectable viral load	-	-	**3.5** [2.0–7.0]	-	-	-
CD4 Nadir (%)	-	**19.6** [9.5–30.7]	**14.5** [8.9–19.9]	-	-	0,301 †
CD4 Nadir (cells count/μl)	-	**380.8** [67–517]	**244.5** [123–349]	-	-	0,681 †
CD4 at sampling (cells count/μl)	**1018**[818–1305]	**454**[54–822]	**757**[514–895]	<0,001* ‡	0,014* ‡	0,179 ‡
% of B cells at sampling	**10,3**[9,0–11,8]	**6,0**[4,1–8,8]	**8,7**[6,2–13,2]	0,022* ‡	1,000 ‡	0,038* ‡
B cells at sampling (cells count/μl)	**231**[177–354]	**83**[39–162]	**185**[136–421]	0,027* ‡	1,000 ‡	0,016* ‡

(a) Control: healthy individuals; (b) Naïve: HIV+ treatment-naïve individuals; (c) Treated: treated-HIV+ individuals

^†^ Mann-Whitney test

^‡^ Mann-Whitney test with Bonferroni Correction

* p<0,05

ND: not detectable. Q1: quartile 1. Q3: quartile 3

HIV^+^ treatment-naïve individuals were recruited at the time of diagnosis just before starting antiretroviral treatment. They showed a median of plasma VL of 6.31x10^5^ copies/ml (1.000–1.258.925; IQR). Treated-HIV^+^ individuals had no detectable plasma VL (<50 copies/mL) for an average of 3.5 years (2.0–7.0; IQR). Treatment-naïve and treated-HIV^+^ individuals showed the same levels of nadir CD4 T cell count (380.8 cells/μl [67–517] IQR and 244.5 cells/μl [123–239] IQR, respectively, *p* = 0.681) and % of nadir CD4 (19.6% [9.5–30.7] IQR and 14.5% [8.9–19.9] IQR, respectively, *p* = 0.301). The absolute CD4 T cell numbers at sampling were greatly and significantly decreased in the naïve and treated-HIV^+^ individuals in comparison to the healthy individuals (Table 1). The frequency and absolute B cell numbers at sampling also decreased in naïve individuals in comparison to treated-HIV^+^ individuals and healthy individuals (Table 1).

### Dysregulation of Treg compartment in viremic HIV^+^ patients

**Fig 1:**
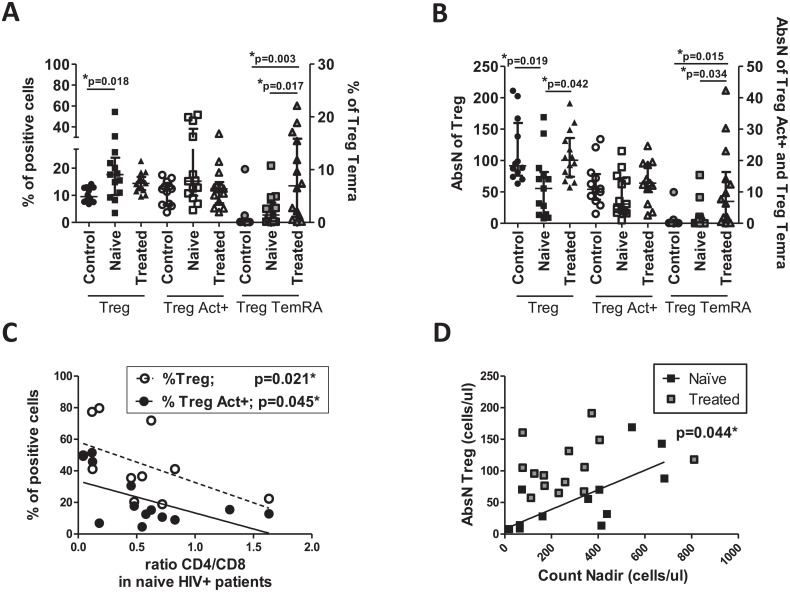
Alteration of Treg compartments in HIV+ and healthy individuals. (A) Frequencies and (B) absolute numbers (AbsN) of Treg, activated Treg (Treg Act^+^: CD25^+^CD127^neg^HLA-DR^+^CD45RO^+^) and Treg TemRA (CD25^+^CD127^neg^CD27^neg^CD45RA^+^) were determined in whole blood using cell surface labeling. Frequencies of total Treg were obtained relative to CD4^+^ T cells and frequencies of Treg subsets were obtained relative to CD25^+^CD127^neg^. (C) Correlation between frequencies of total Treg or Treg Act^+^ and the ratio CD4/CD8 in HIV^+^ treatment-naïve individuals. (D) Correlation between absolute counts of Treg (AbsN Treg) and nadir count (black squares: HIV^+^ treatment-naïve individuals and grey squares: treated-HIV^+^ individuals). Medians ± IQRs are represented. Correlations were determined by Spearman’s rank correlation. Control: Healthy individuals. Naïve: HIV+ treatment-naïve individuals. Treated: Treated-HIV+ individuals. * = p<0.05 when comparing conditions. Each symbol corresponds to an individual.

We investigated the presence of Treg (CD4^+^CD25^+^CD127^neg^) in HIV^+^ patients (S1A Fig) and compared to the frequency, the absolute number of Treg cells was significantly decreased in HIV^+^ treatment-naïve individuals (Fig 1A), as expected. No differences were observed in frequency or counts of activated Treg between groups. However, the percentage and counts of the Treg TemRA were increased in treated-HIV^+^ individuals (S1A Fig and Fig 1B).

Interestingly, the increase of Treg (and activated Treg) frequencies in HIV^+^ treatment-naïve individuals correlated negatively with the CD4/CD8 ratio (Fig 1C), perhaps in an attempt to reestablish the T-cell homeostasis lost during HIV infection (CD4^+^ T-cell depletion (Table 1) and CD8^+^ T-cell expansion (*data not shown*). Moreover, Treg cellular counts in HIV^+^ individuals were also positively correlated with nadir count, which is related to the disease progression (Fig 1D). Effective anti-HIV treatment enabled a reversion of the decrease in cell count of total Treg but did not allow the reversion of Treg TemRA levels (Fig 1B). Therefore, T-cell populations were undergoing profound changes that were not all reversed with the use of the treatment.

### Dysregulation of Breg compartment in viremic HIV^+^ patients

**Fig 2:**
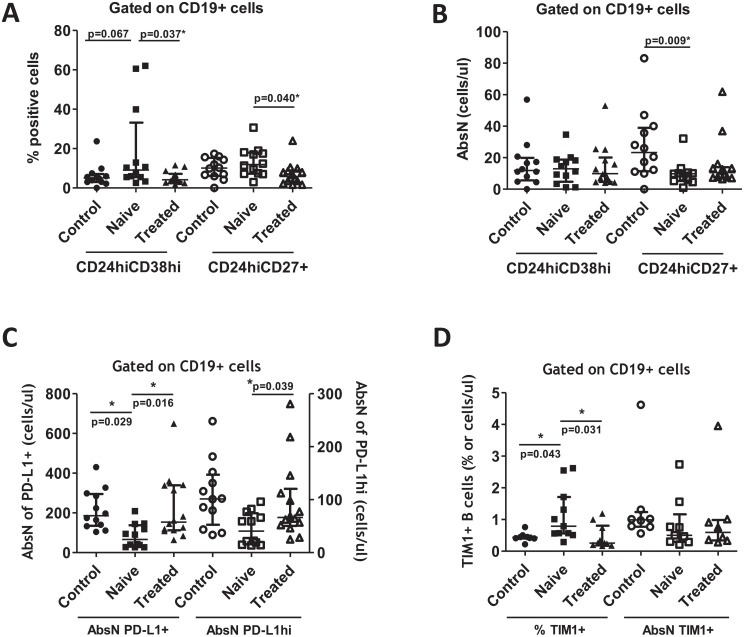
Breg compartments in HIV+ and healthy individuals. (A) Frequencies and (B) absolute numbers (AbsN) of CD24^hi^CD38^hi^ and CD24^hi^CD27^+^ Breg subsets. (C) Absolute numbers of PD-L1^+^ and PD-L1^hi^ B cells. (D) Frequencies and absolute numbers of TIM-1^+^ B cells. All of these subsets were determined in whole blood using cell surface labeling. Frequencies of Breg were obtained relative to CD19^+^ cells. Medians ± IQRs are represented. Control: Healthy individuals. Naïve: HIV+ treatment-naïve individuals. Treated: Treated-HIV+ individuals. * = p<0.05 when comparing conditions. Each symbol corresponds to an individual.

The Breg subsets exert a regulatory function that suppresses pathogenic T cells and helps in the maintenance of the Treg. We studied the presence of various Breg subsets (S2 Fig). The frequencies of the CD19^+^CD24^hi^CD27^+^ and CD19^+^CD24^hi^CD38^hi^ subsets were increased in HIV^+^ treatment-naïve individuals in comparison with healthy individuals, and effective treatment induced a significant decrease of Breg frequencies in treated-HIV^+^ individuals (Fig 2A). Interestingly, the absolute counts of CD19^+^CD24^hi^CD27^+^ were significantly decreased in HIV^+^ treatment-naïve individuals in comparison to healthy individuals (Fig 2B). Other B-cell subsets are considered as regulatory B cells, and we previously demonstrated that the frequencies of PD-L1^+^ and PD-L1^hi^ in CD19^+^ cells were significantly increased in the same cohorts of HIV^+^ and healthy individuals [30]. We showed that the absolute counts of these cells (PD-L1^+^ and PD-L1^hi^ in CD19^+^ cells) were significantly decreased in HIV^+^ treatment-naïve individuals in comparison to treated-HIV^+^ or healthy individuals (Fig 2C). Furthermore, we also showed that the frequency of CD19^+^TIM-1^+^ was increased in HIV^+^ treatment-naïve individuals, but not the absolute counts (Fig 2D). Thus, the Treg compartment was dysregulated by the presence of HIV, but also the Breg compartment. Breg subsets presented a general increase in frequencies and a general decrease in absolute counts in HIV^+^ treatment-naïve individuals in comparison to treated-HIV^+^ or healthy individuals.

### IL-10-production in B cells and Breg subsets

The determination of Breg by cell surface markers must be performed with care since it has been demonstrated that HIV can directly or indirectly modify the expression of surface markers such as CD27 [20, 30–32]. IL-10 has an anti-inflammatory and suppressive effect on most hematopoietic cells. Thus, it is considered as the principal marker for Breg functionality in peripheral blood.

**Fig 3:**
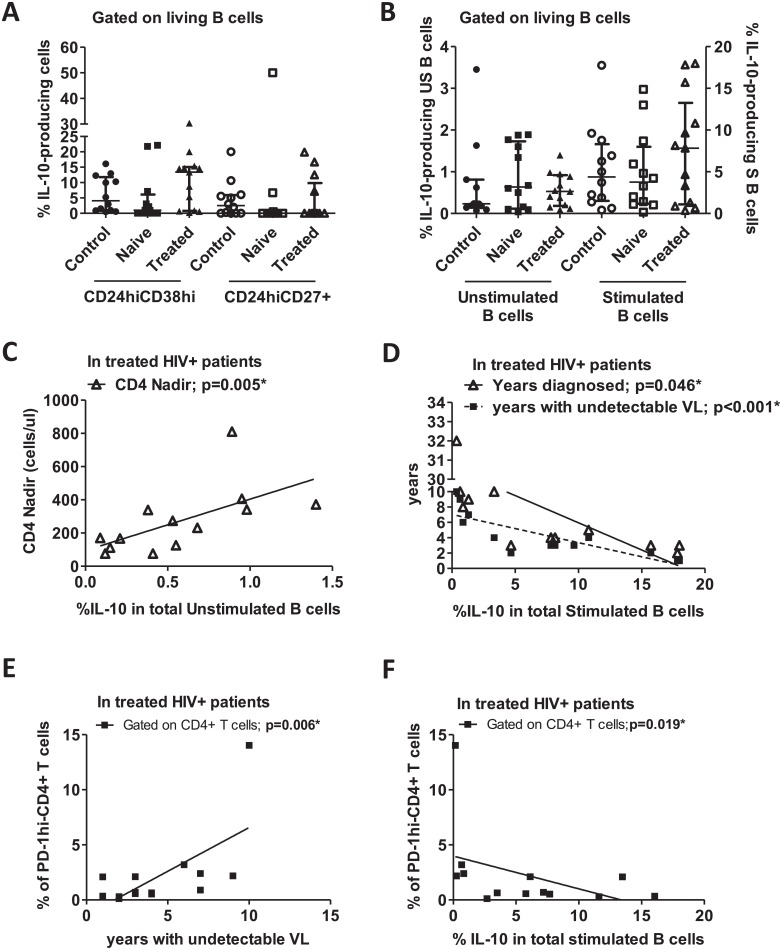
IL-10-producing Breg subsets in HIV^+^ patients and healthy individuals. PBMCs were isolated, and some cells were stimulated with CD40L/LPS/CpG. After 2 days, cells were labeled for surface markers and intracellular IL-10. (A) Frequencies of IL-10-producing-CD19^+^CD24^hi^CD38^hi^ and CD19^+^CD24^hi^CD27^+^; (B) Frequencies of unstimulated (US) and stimulated (S) total IL-10-producing B cells. Frequencies were obtained with gating on CD19^+^ cells. (C) Correlation between basal expressions of IL-10 (frequency of IL-10-producing B cells without stimulation) and CD4 nadir count in treated-HIV^+^ individuals. (D) Correlation between frequencies of IL-10-producing B cells and years diagnosed or years with undetectable VL in treated-HIV^+^ individuals. (E) Correlation between frequencies of PD-1^hi^-CD4^+^ T cells and years with undetectable VL in treated-HIV^+^ individuals. (F) Correlation between frequencies of PD-1^hi^-CD4^+^ T cells and frequencies of IL-10-producing stimulated B cells in treated-HIV^+^ individuals. Medians ± IQRs are represented. Correlations were determined by Spearman’s rank correlation. Control: Healthy individuals. Naïve: HIV^+^ treatment-naïve individuals. Treated: Treated-HIV^+^ individuals. * = p<0.05. Each symbol corresponds to an individual.

PBMCs were isolated from peripheral blood and stimulated for 48h with CpG, CD40L and LPS, and these activators together induce the Breg phenotype. We quantified the frequency of IL-10-producing cells gated on Breg subsets (CD19^+^CD24^hi^CD38^hi^ and CD19^+^CD24^hi^CD27^+^, S3 Fig) and on total B cells (CD19^+^) since the use of phenotypic markers must be challenged in the context of HIV. No changes were observed in the IL-10-producing CD19^+^CD24^hi^CD38^hi^ or CD19^+^CD24^hi^CD27^+^ Breg subsets (Fig 3A) and in total stimulated or unstimulated B cells (Fig 3B). The IL-10 detected in unstimulated B cells represents the basal level of IL-10-B-cell production.

In treated-HIV^+^ individuals, we distinguished two groups of patients: those that expressed high frequency of intracellular IL-10 in B cells, and those that did not express intracellular IL-10 or only slightly expressed it (Fig 3A and 3B). Hence, we analyzed their relationship with other markers, and a correlation was observed between the IL-10-producing unstimulated total B cells and CD4 nadir count in treated-HIV^+^ individuals (Fig 3C). Therefore, the basal ability of total B cells to produce IL-10 could possibly be related to disease progression since a low CD4 nadir count is associated with upcoming outcomes in the course of HIV disease [33, 34].

Moreover, in treated-HIV^+^ individuals, we found a negative correlation between the frequency of the total stimulated IL-10-producing B cells and the years diagnosed or the years with undetectable VL (Fig 3D). A correlation was also observed between years with undetectable VL and the frequency of CD4^+^ T cells expressing PD-1, an exhaustion marker that is related to the impairment of the CD4^+^ T cell population over time (Fig 3E, S1B Fig). Moreover, the same marker was negatively correlated with the frequency of stimulated IL-10-producing B cells (Fig 3F). Therefore, the loss of IL-10 production in stimulated B cells in treated-HIV^+^ individuals was associated with CD4^+^ T-cell exhaustion during HIV infection in the absence of viral replication.

### Loss of T-cell proliferation ability in HIV^+^ patients

**Fig 4:**
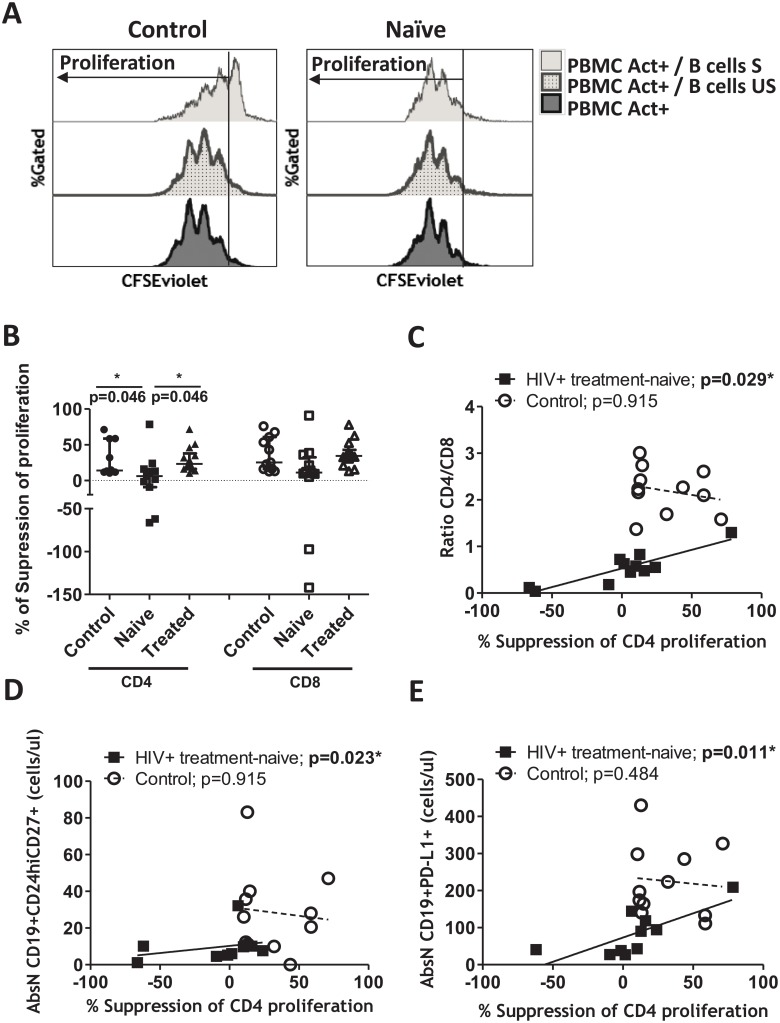
Suppression of PBMC proliferation. B cells were isolated and stimulated with CD40L/LPS/CpG for 2 days. Unstimulated (US) and stimulated (S) B cells were then washed and co-cultured with autologous CFSE-labeled PBMCs for 3 days. (A) Proliferation was followed in living cells as the loss of CFSE signal. Histogram plots showing the PBMC proliferation under anti-CD3/anti-CD28 activation, alone (PBMC Act^+^) or co-cultured with S or US B cells. Histograms representing one healthy (Control) and one HIV^+^ treatment-naïve individuals (Naïve) are shown. (B) Percentage of suppression of proliferation of CD4^+^ or CD8^+^ T cells was calculated as follows: (100-(proliferation of CFSE-labeled T cells in co-culture with stimulated B cells*100/ proliferation of CFSE-labeled T cells in co-culture with unstimulated B cells)). Negative values corresponded to an increase in proliferation. Medians ± IQRs are represented. (C) Correlation between percentage of suppression of proliferation of CD4^+^ T cells and CD4/CD8 ratio in HIV+ treatment-naïve and healthy individuals. (D) Correlation between percentage of suppression of proliferation of CD4^+^ T cells and absolute numbers (AbsN) of CD19^+^CD24^hi^CD27^+^ in HIV^+^ treatment-naïve (Naïve) and healthy individuals. (E) Correlation between percentage of suppression of proliferation of CD4^+^ T cells and absolute numbers (AbsN) of CD19^+^PD-L1^+^ in HIV^+^ treatment-naïve (Naïve) and healthy individuals. Correlations were determined by Spearman’s rank correlation. * = p<0.05 when comparing conditions. Each symbol corresponds to an individual.

To determine whether Breg cells conserved their function, B cells isolated from PBMCs were stimulated with CD40L/LPS/CpG for two days. This mix of activators is known to activate the Breg subsets [7]. Unstimulated B cells from the same individuals were used as a control. After two days, unstimulated and stimulated B cells were co-cultured with autologous CFSE-labeled PBMCs to observe the ability of the activated Breg to suppress the proliferation of the CFSE-PBMCs (Fig 4A).

The suppressive capacity of stimulated B cells (condition of Breg induction) over CD4^+^ and CD8^+^ T cell proliferation was observed and compared with the suppressive ability of unstimulated B cells (non-Breg induction). Unstimulated B cells did not show a suppressive function over PBMC proliferation, as expected (Fig 4A and 4B). In contrast, stimulated B cells from healthy individuals (Control) showed the ability to limit PBMCs proliferation (Fig 4A). On the other hand, the capacity of stimulated B cells to restrict the CFSE-CD4^+^ T-cell proliferation was lost in HIV^+^ treatment-naïve individuals compared to treated-HIV^+^ or healthy controls (Fig 4B). The suppression of the CD8^+^ T-cell proliferation was decreased when co-cultured with stimulated B cells from HIV^+^ treatment-naïve individuals, but the decrease was not significant in comparison to the suppression of the CD4^+^ T-cell proliferation (Fig 4B). Furthermore, increased proliferation was observed in some HIV^+^ treatment-naïve individuals instead of suppression (represented by a negative percentage of suppression in Fig 4B). This suggests that B cells activated with Breg-associated *stimuli* either had utterly lost their suppressive function or that autologous CFSE-T cells were no longer sensitive to the suppressive function of Breg.

Because of the heterogeneity of the suppressive ability over CD4 proliferation observed in HIV^+^ treatment-naïve individuals (Fig 4B), we analyzed the correlations that the suppression of CD4 proliferation have with the CD4/CD8 ratio and the Breg subsets phenotype. Interestingly, there was a positive correlation between the frequency of suppression of CD4 proliferation and the CD4/CD8 ratio in HIV^+^ treatment-naïve individuals (Fig 4C). Moreover, a positive correlation was observed between the frequency of suppression of CD4^+^ T cell proliferation and cell counts of CD24^hi^CD27^+^ Breg subset (Fig 4D) and cell counts of CD19^+^PD-L1^+^ (Fig 4E), but not with the cell counts of CD24^hi^CD38^hi^ Breg subset, or the frequency of the IL-10-producing B cells or with the cell counts of CD19^+^TIM-1^+^ (*data not shown*). In summary, the loss of suppressive ability over CD4^+^ T-cell proliferation is observed in patients with a lower CD4/CD8 ratio and lower CD24^hi^CD27^+^ and CD19^+^PD-L1^+^ Breg-subset counts.

### Breg subsets might be functionally distinct

The IL-10 production is closely linked to Breg function. Since we observed changes in frequency and absolute numbers of several Breg subsets (Fig 2), we analyzed whether there was a correlation between the frequency of Breg subsets and IL-10-producing B cells. The increased presence of CD19^+^CD24^hi^CD27^+^ cells was correlated with a decrease in IL-10-producing B cell frequencies in healthy individuals (Table 2). In contrast, the frequencies of CD19^+^PD-L1^hi^ cells and IL-10-producing B cells were positively correlated in healthy individuals. These correlations were lost in HIV^+^ treatment-naïve individuals, which could suggest that the fine-tuned regulation between IL-10-producing B cells and Breg subsets might be lost in HIV^+^ individuals.

**Table 2:** Correlations between frequencies of Breg subsets and IL-10-producing B cells and Treg.

		Control (a)		Naive (b)		Treated (c)	
Condition 1(d)	Condition 2	Coefficient	p	Coefficient	p	Coefficient	p
% CD24^hi^CD38^hi^	% IL-10-producing CD19^+^	-0,322	0,308	0,323	0,332	-0,357	0,231
% CD24^hi^CD27^+^	% IL-10-producing CD19^+^	-0,804	0,002*	-0,036	0,915	0,270	0,373
% PD-L1^hi^	% IL-10-producing CD19^+^	0,802	0,002*	-0,136	0,689	-0,231	0,448
% TIM-1^+^	% IL-10-producing CD19^+^	0,168	0,691	0,692	0,018*	-0,830	0,003*
% CD24^hi^CD38^hi^	% Treg (%CD25^+^CD127^neg^)	0,262	0,411	0,860	<0,001*	0,086	0,771
% CD24^hi^CD38^hi^	% CD25^+^Foxp3^+^	0,041	0,898	0,851	<0,001*	0,218	0,455
% CD24^hi^CD27^+^	% Treg (%CD25^+^CD127^neg^)	0,385	0,217	-0,196	0,542	-0,275	0,341
% CD24^hi^CD27^+^	% CD25^+^Foxp3^+^	-0,032	0,923	-0,259	0,417	-0,328	0,253
% PD-L1^hi^	% Treg (%CD25^+^CD127^neg^)	-0,070	0,829	-0,259	0,417	0,125	0,670
% PD-L1^hi^	% CD25^+^Foxp3^+^	0,389	0,212	-0,014	0,966	0,266	0,358
% TIM-1^+^	% Treg (%CD25^+^CD127^neg^)	-0,144	0,734	0,251	0,457	-0,067	0,855
% TIM-1^+^	% CD25^+^Foxp3^+^	-0,422	0,298	0,656	0,028*	0,515	0,128

(a) Control: healthy individuals; (b) Naïve: treatment-naïve HIV^+^ patients. (c) Treated: treated HIV^+^ patients. (d) Gated on CD19+ cells.

Frequency of Treg (CD25^+^CD127^neg^, frequency calculated relative to CD4+ T cells) or CD25^+^Foxp3^+^ (frequency calculated relative to CD4^+^ T cells) were determined on whole blood or on isolated PBMC, respectively.

Correlations were determined by Spearman’s rank correlation.

Coefficient: Spearman correlation coefficient.

*p<0,05

On the other hand, the frequencies of CD19^+^CD24^hi^CD38^hi^ and CD19^+^TIM-1^+^ subsets did not show correlation with IL-10-producing B cell subsets in healthy individuals (Control). Interestingly, the frequency of the CD19^+^TIM-1^+^ subset presented a significant correlation with IL-10-producing B cells in HIV^+^ individuals.

Since some Breg subsets were described to influence Treg differentiation, we further analyzed the possible correlations between various subsets of the Breg and Treg compartments (CD4^+^CD25^+^CD127^neg^ and CD4^+^CD25^+^Foxp3^+^). Positive correlations between frequencies of Breg (CD24^hi^CD38^hi^) or CD19^+^TIM-1^+^ and Treg in HIV^+^ treatment-naïve individuals were observed (Table 2). However, these correlations were not observed in healthy individuals (Control) or in treated-HIV^+^ individuals. It is interesting to note that frequencies of CD19^+^CD24^hi^CD27^+^ or CD19^+^PD-L1^+^ subsets, which were correlated with the frequency of IL-10-producing of B cells, did not present any correlation with Treg (Table 2). Thus, these four Breg subsets cannot be functionally related since they presented different correlations with the B-cell function such as the IL-10 production or with the frequency of Treg, which are two important markers in the regulation of the immune system during HIV infection.

## Discussion

To our knowledge, this is the first time that the relationships between regulatory immune cell compartments where a general dysregulation of phenotype and function occur have been defined. We observed no changes or slight increases in the frequency of the Breg subsets and decreases in the absolute counts in HIV^+^ treatment-naïve individuals in comparison to healthy individuals. No changes were observed in the frequency of IL-10-producing Breg subsets or total IL-10-producing B cells. Our results differ from previous works that described an increase in IL-10-producing total B cells [35] and IL-10-producing CD19^+^CD24^hi^CD38^hi^ [16, 19, 35]. However, the major difference in our work is that we studied IL-10 intracellular expression in PBMCs stimulated with a mix of stimuli (CD40L, CpG and LPS) that induces a Breg phenotype. This contrasts with other works where the labeling was performed directly on freshly isolated PBMCs stimulated with PMA+ionomycin [18, 19, 35]. Another study on common variable immunodeficiency showed that Breg were defective in IL-10 expression and that the frequency of IL-10-producing CD24^hi^CD38^hi^ or CD24^hi^CD27^+^ B cells was decreased when the cell culture contained CpG [36]. Therefore, the induction of IL-10 production by Breg is highly dependent on the type of stimulation used.

During HIV infection, the total B-cell compartment shows dysregulated counts and function via contact with the T lymphocytes or viral particles [20, 31, 37]. We previously demonstrated that HIV by itself is able to dysregulate B cells *in vitro* through a Breg-like phenotype [20]. Moreover, a low proportion of IL-10-producing B cells was observed in viremic controller individuals in comparison to viremic individuals [38]. Therefore, we hypothesized that the presence of viral replication could increase the IL-10-producing cells, although the mechanism is not yet known. In our work, we did not observe a change in the level of IL-10 production in stimulated or un-stimulated B cells in HIV^+^ or healthy individuals. However, IL-10 production was negatively correlated with exhausted CD4^+^ T cells (expressing PD-1) and years of undetectable VL in HIV^+^ treated individuals. This shows that even when successfully treated, years of HIV infection could be related to the IL-10 production ability and CD4^+^ T-cell exhaustion. Because it has been hypothesized that the Breg compartment might be dysregulated as a consequence of chronic inflammation in autoimmune diseases [5], it will be of great interest in future research to examine the loss of Breg functionality and to verify the level of Breg activation and exhaustion during HIV infection.

We observed an increase in the frequencies of the CD24^hi^CD27^+^ and PD-L1^+^ Breg subsets in HIV^+^ treatment-naïve individuals. These subsets are related to a decrease of suppressive function and the ability to control the IL-10 production of B cells (Table 2). PD-L1^hi^-Breg are not the major IL-10 producer but might act through an alternative suppressive mechanism [10]. IL-10 and PD-L1 are induced during cellular activation [39], and thus, the question arises of whether they are not produced by the same mechanisms of cellular activation or PD-L1^hi^ and IL-10 expression might be controlled by the same pathway. Since IL-10 is produced after cellular activation, one can hypothesize that CD24^hi^CD27^+^ Breg could negatively regulate the production of IL-10 from activated B cells in healthy individuals (Table 2). Therefore, dysregulation of the CD24^hi^CD27^+^ and CD19^+^PD-L1^+^ Breg subsets could act through different mechanisms, namely dysregulation of the IL-10 production and dysregulation of their suppressive ability during cellular activation. We could not conclude that both mechanisms were linked in this study, and further research must be performed to clarify this pivotal point.

We demonstrated that CD19^+^TIM-1^+^ could also participate in the IL-10 production in HIV^+^ individuals, which has already been described in mice, where TIM-1 defects in B cells reduce the production of Breg IL-10 [40]. Moreover, it was demonstrated that IL-10-secreting B cells were concentrated in the TIM-1^+^CD19^+^ B cells in healthy [41] and in HIV^+^ individuals [19]. This IL-10 production by TIM-1^+^ B cells is related to their ability to inhibit IFN-γ production and to upregulate Foxp3 expression in T cells [41]. TIM-1^+^ B cells were also determined to promote Foxp3 expression in CD4+ T cells in acute respiratory distress syndrome [42]. We demonstrated an increase in the frequency of TIM-1^+^ B cells in the HIV^+^ treatment-naïve patients, which could be related to the observed increase in the Treg frequency (Table 2). We did not quantify the IL-10 production of TIM-1^+^ B cells, but it would be interesting to examine whether the modulation of Treg frequency by TIM-1^+^ Breg could be done through IL-10 production.

CD19^+^CD24^hi^CD38^hi^ levels did not present a correlation with the suppression of T cell proliferation, or with cellular ability to produce IL-10, which leads us to suggest that this Breg subset can have a distinct role during HIV infection. CD19^+^CD24^hi^CD38^hi^ and CD19^+^TIM-1^+^ frequencies were positively correlated with the frequency of Treg in HIV^+^ treatment-naïve individuals, and this relation was lost in treated-HIV^+^ and healthy individuals (Control). It has been demonstrated that CD19^+^CD24^hi^CD38^hi^ cells induce the conversion of CD4^+^CD25^neg^ into Treg [43, 44]. Indeed, in pathogenic conditions such as rheumatoid arthritis, reduced frequency of CD19^+^CD24^hi^CD38^hi^ cells was related to a limited ability to induce Treg [43]. Such a relation was also demonstrated in cancer where an increased level of CD19^+^CD24^hi^CD38^hi^ cells was directly correlated with the increased frequency of circulating Treg in breast and gastric cancers [45, 46]. Dysregulation of CD24^hi^CD38^hi^ and TIM-1^+^ Breg subsets such as increased frequencies might act on Treg cell counts and function in the context of HIV. Therefore, CD19^+^CD24^hi^CD27^+^ and CD19^+^PD-L1^+^ could participate in the IL-10 production and suppressive function in CD4^+^ T cell proliferation and CD19^+^CD24^hi^CD38^hi^ and CD19^+^TIM-1^+^ could be related to Treg homeostasis. In this context, it was already observed that some factors such as B Cell-Activating Factor (BAFF), responsible for the B-cell maturation and survival, were elevated and associated with progressive diseases in HIV^+^ individuals [47, 48]. BAFF is as well a factor that was related to a high level of IL-10 [48], IL-10-producing B-cell frequency [49, 50] and Treg differentiation in mice models [51]. Therefore, IL-10-producing B cells and expansion of Treg have been related to the expression of BAFF. However, more recent studies demonstrate that even if BAFF alone in *in vitro* and *in vivo* models was able to induce the frequency of IL-10-expressing Breg, BAFF selects IL-10^neg^ B cells over IL-10^+^ B cells during inflammatory responses [52], and BAFF does not promote IL-10 on CpG-activated B cells [53]. Because inflammation is pivotal in the HIV context, these last results could explain why we did not observe an increase in IL-10-producing B-cell frequencies in HIV^+^ individuals. Thus, it will be very interesting to analyze if BAFF is implicated in the dysregulation of Breg and Treg compartments during HIV infection in further work. Therefore, further studies with larger sample sizes or patients could help to confirm these hypotheses. Indeed, the modest sample size is one of the limitations of our study, and the population studied was predominantly male (>75%), so generalization to females should also be done cautiously.

Our study suggests that various Breg subsets could have different functions during B and T cell homeostasis during HIV-1 infection. Moreover, the dysregulation of Breg and Treg compartments was reverted in part by effective anti-retroviral therapy, and interrelation between these two groups of immunosuppressive cells was deeply perturbed during HIV infection. Therefore, Breg and Treg might be unable to respond adequately to HIV infection.

## Supporting information

S1 Fig Dot plots defining CD4^+^ T, CD8^+^ T, Treg, and exhausted CD4+ T cells. Whole blood was labeled to determine (A) the CD4^+^ T cells (gated on lymphocyte population). Frequency of activated CD4^+^ T cells defined as CD45RO^+^HLA-DR^+^ was determined relative to the CD4^+^ gate. Treg were determined as CD127^neg^CD25^+^. Activated Treg and Treg TemRA were determined as CD45RO^+^HLA-DR^+^ and CD27^neg^CD45RA^+^, respectively, gated on the CD4^+^CD127^neg^CD25^+^ compartment. Frequencies of Treg and Treg subsets were determined relative to the CD4^+^CD127^neg^CD25^+^ gate. (B) Frequency of exhausted CD4^+^ T cells defined as PD-1^+^ and PD-1^hi^ was determined relative to the CD4^+^ gate. Dot plots from one donor are shown.
(PPTX)

S2 Fig Dot plot defining the different Breg subsets. Whole blood was labeled to determine (A) the frequency of B cells (CD19^+^, gated on lymphocyte population). (B) Frequencies of four Breg subsets gated on B cells (CD19^+^ cells) such as (i) CD24^hi^CD38^hi^, (ii) CD24^hi^CD27^+^, (iii) TIM-1^+^ B cells and (iv) PD-L1^+^ and PD-L1^hi^ B cells. Dot plots from one donor are shown.
(PPTX)

S3 Fig Phenotype definition of Breg and IL-10-producing B cells. **(A)** Isolated PBMCs were stimulated for 2 days and labeled to determine the living cell population. (B) Total B cells gated on living lymphocytes and (C) Breg subsets gated on total B cells (CD19^+^ cells) such as (i) CD24^hi^CD38^hi^ and (ii) CD24^hi^CD27^+^ were determined. (D) Frequencies of IL-10-producing total B cells and (E) IL-10-producing Breg such as (i) CD24^hi^CD38^hi^ and (ii) CD24^hi^CD27^+^ were quantified. Dot plots from one donor are shown.
(PPTX)

S1 Table Flow cytometry panels. VD: Viability dye. CAL: Calibration beads to quantify absolute cell counts. (a) Beckman-Coulter. (b) BioLegend. (c) BD Biosciences. (d) Immunological Sciences. (e) Miltenyi Biotec. (f) eBioscience.
(PPTX)

S2 Table Cellular populations followed in this study. Description of the T and B-cell subsets followed in this study and the gating strategies.
(PPTX)
