# Supplementary figures and images for: Analysis of the dysregulation between regulatory B and T cells (Breg and Treg) in human immunodeficiency virus (HIV)-infected patients
Source: PLoS One. 2019 Mar 27;14(3):e0213744. doi: 10.1371/journal.pone.0213744 (PMC6436717; doi:10.1371/journal.pone.0213744)

## Slide 1
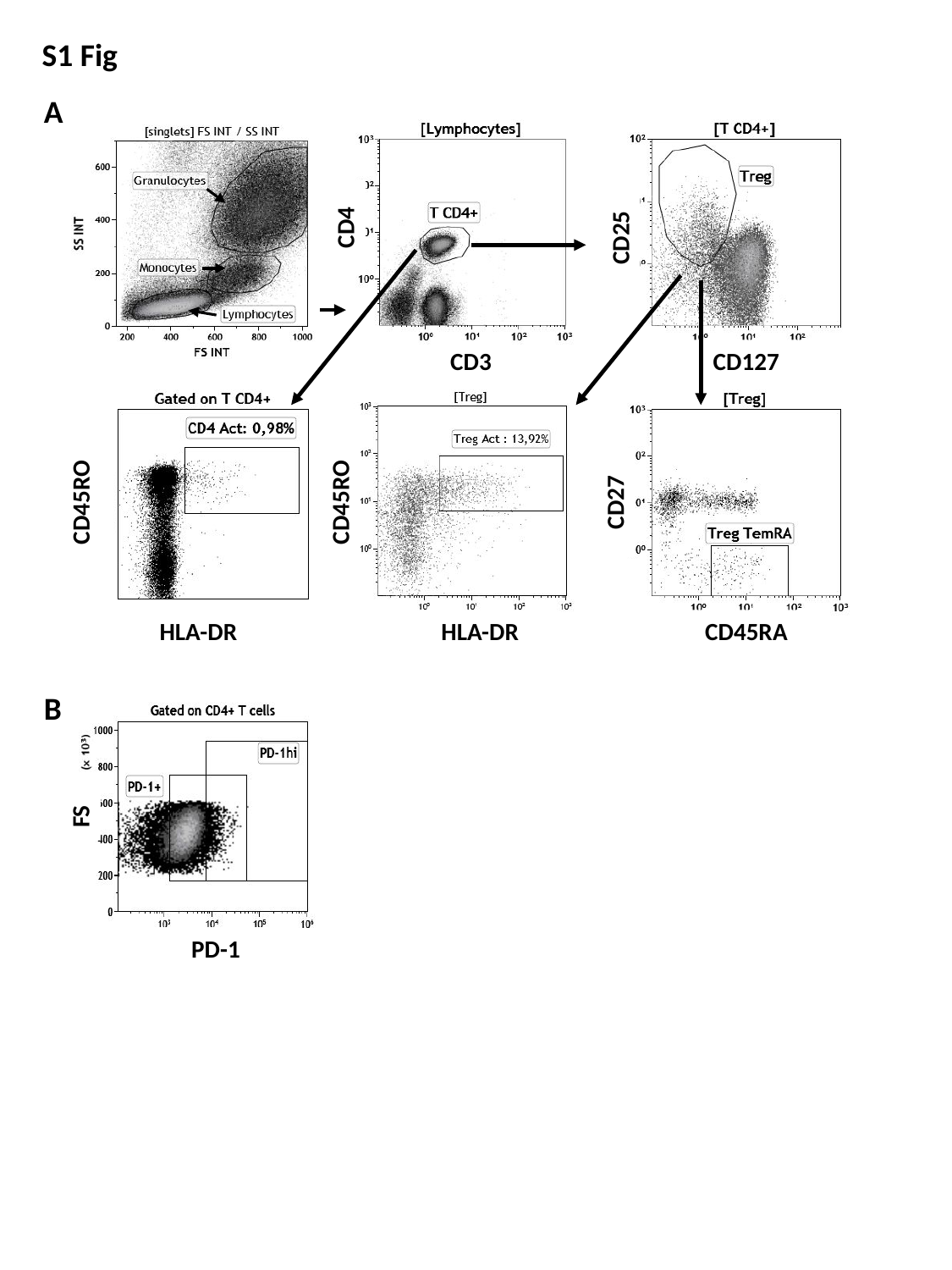

S1 Fig
A
CD4
CD25
CD3
CD127
CD45RO
CD45RO
CD27
HLA-DR
HLA-DR
CD45RA
B
FS
PD-1

Supplement: S1 Fig — Whole blood was labeled to determine (A) the CD4+ T cells (gated on lymphocyte population). Frequency of activated CD4+ T cells defined as CD45RO+HLA-DR+ was determined relative to the CD4+ gate. Treg were determined as CD127negCD25+. Activated Treg and Treg TemRA were determined as CD45RO+HLA-DR+ and CD27negCD45RA+, respectively, gated on the CD4+CD127negCD25+ compartment. Frequencies of Treg and Treg subsets were determined relative to the CD4+CD127negCD25+ gate. (B) Frequency of exhausted CD4+ T cells defined as PD-1+ and PD-1hi was determined relative to the CD4+ gate. Dot plots from one donor are shown. (PPTX) [file pone.0213744.s001.pptx]
