# Supplementary material for: Analysis of the dysregulation between regulatory B and T cells (Breg and Treg) in human immunodeficiency virus (HIV)-infected patients
Source: PLoS One. 2019 Mar 27;14(3):e0213744. doi: 10.1371/journal.pone.0213744 (PMC6436717; doi:10.1371/journal.pone.0213744)
Supplement: S2 Fig — Whole blood was labeled to determine (A) the frequency of B cells (CD19+, gated on lymphocyte population). (B) Frequencies of four Breg subsets gated on B cells (CD19+ cells) such as (i) CD24hiCD38hi, (ii) CD24hiCD27+, (iii) TIM-1+ B cells and (iv) PD-L1+ and PD-L1hi B cells. Dot plots from one donor are shown. (PPTX) [file pone.0213744.s002.pptx]

## Slide 1
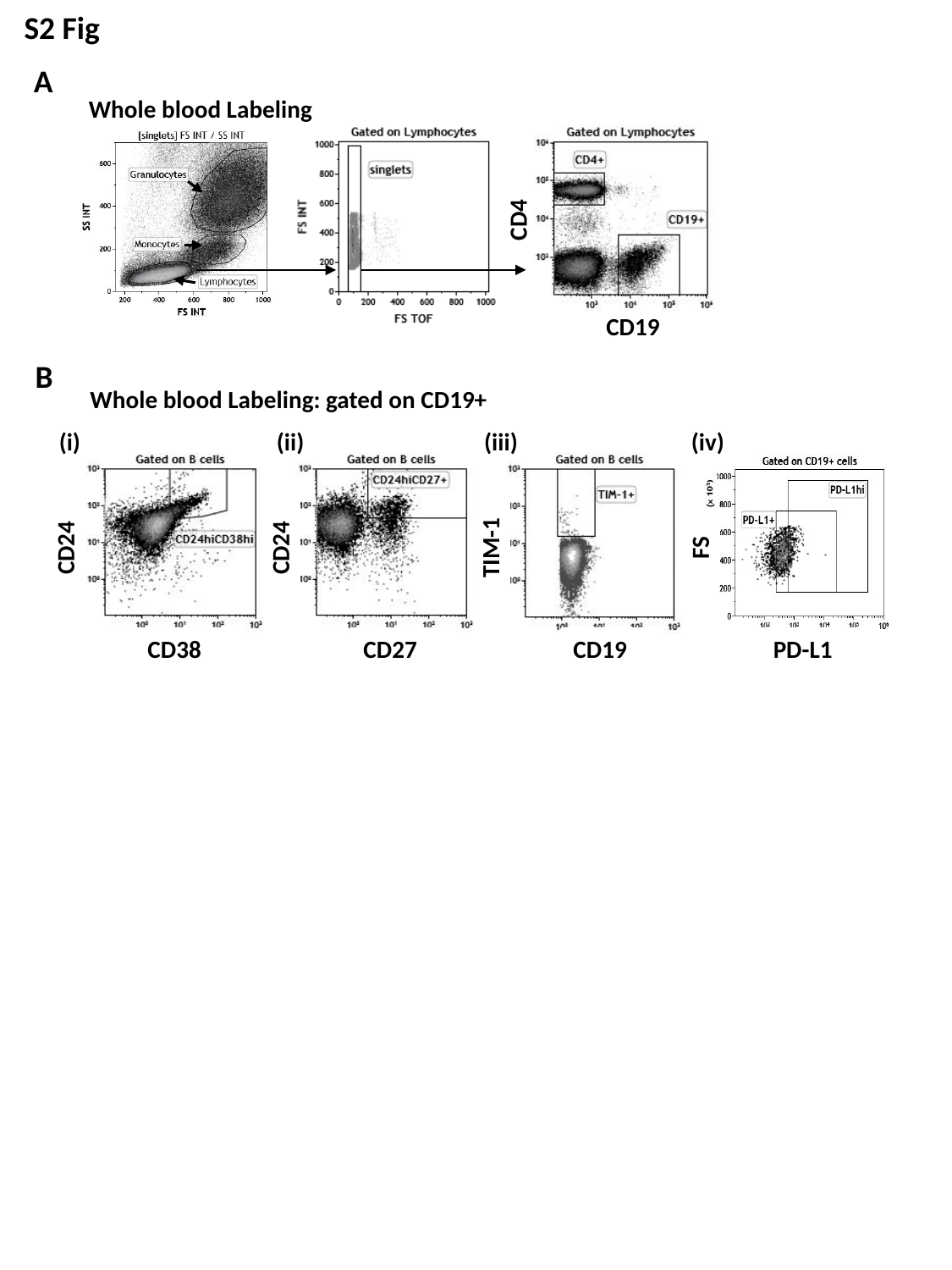

S2 Fig
A
Whole blood Labeling
CD4
CD19
B
Whole blood Labeling: gated on CD19+
(i)
(ii)
(iii)
(iv)
CD24
CD24
TIM-1
FS
CD38
CD27
CD19
PD-L1
