# Supplementary material for: Analysis of the dysregulation between regulatory B and T cells (Breg and Treg) in human immunodeficiency virus (HIV)-infected patients
Source: PLoS One. 2019 Mar 27;14(3):e0213744. doi: 10.1371/journal.pone.0213744 (PMC6436717; doi:10.1371/journal.pone.0213744)
Supplement: S3 Fig — (A) Isolated PBMCs were stimulated for 2 days and labeled to determine the living cell population. (B) Total B cells gated on living lymphocytes and (C) Breg subsets gated on total B cells (CD19+ cells) such as (i) CD24hiCD38hi and (ii) CD24hiCD27+ were determined. (D) Frequencies of IL-10-producing total B cells and (E) IL-10-producing Breg such as (i) CD24hiCD38hi and (ii) CD24hiCD27+ were quantified. Dot plots from one donor are shown. (PPTX) [file pone.0213744.s003.pptx]

## Slide 1
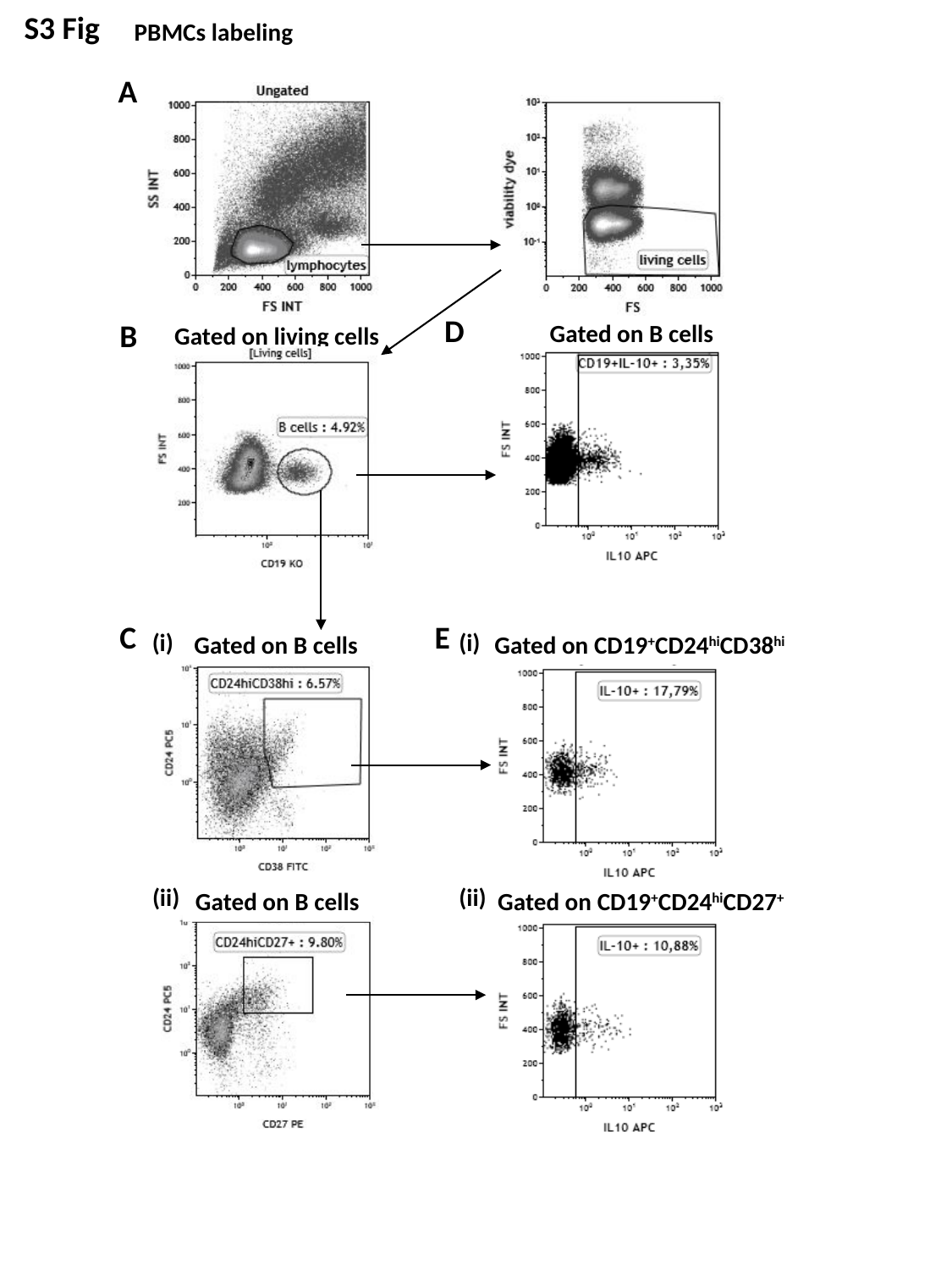

S3 Fig
PBMCs labeling
A
D
B
Gated on B cells
Gated on living cells
C
E
(i)
(i)
Gated on B cells
Gated on CD19+CD24hiCD38hi
(ii)
(ii)
Gated on B cells
Gated on CD19+CD24hiCD27+
