# Supplementary material for: Analysis of the dysregulation between regulatory B and T cells (Breg and Treg) in human immunodeficiency virus (HIV)-infected patients
Source: PLoS One. 2019 Mar 27;14(3):e0213744. doi: 10.1371/journal.pone.0213744 (PMC6436717; doi:10.1371/journal.pone.0213744)
Supplement: S1 Table — VD: Viability dye. CAL: Calibration beads to quantify absolute cell counts. (a) Beckman-Coulter. (b) BioLegend. (c) BD Biosciences. (d) Immunological Sciences. (e) Miltenyi Biotec. (f) eBioscience. (PPTX) [file pone.0213744.s004.pptx]

## Slide 1
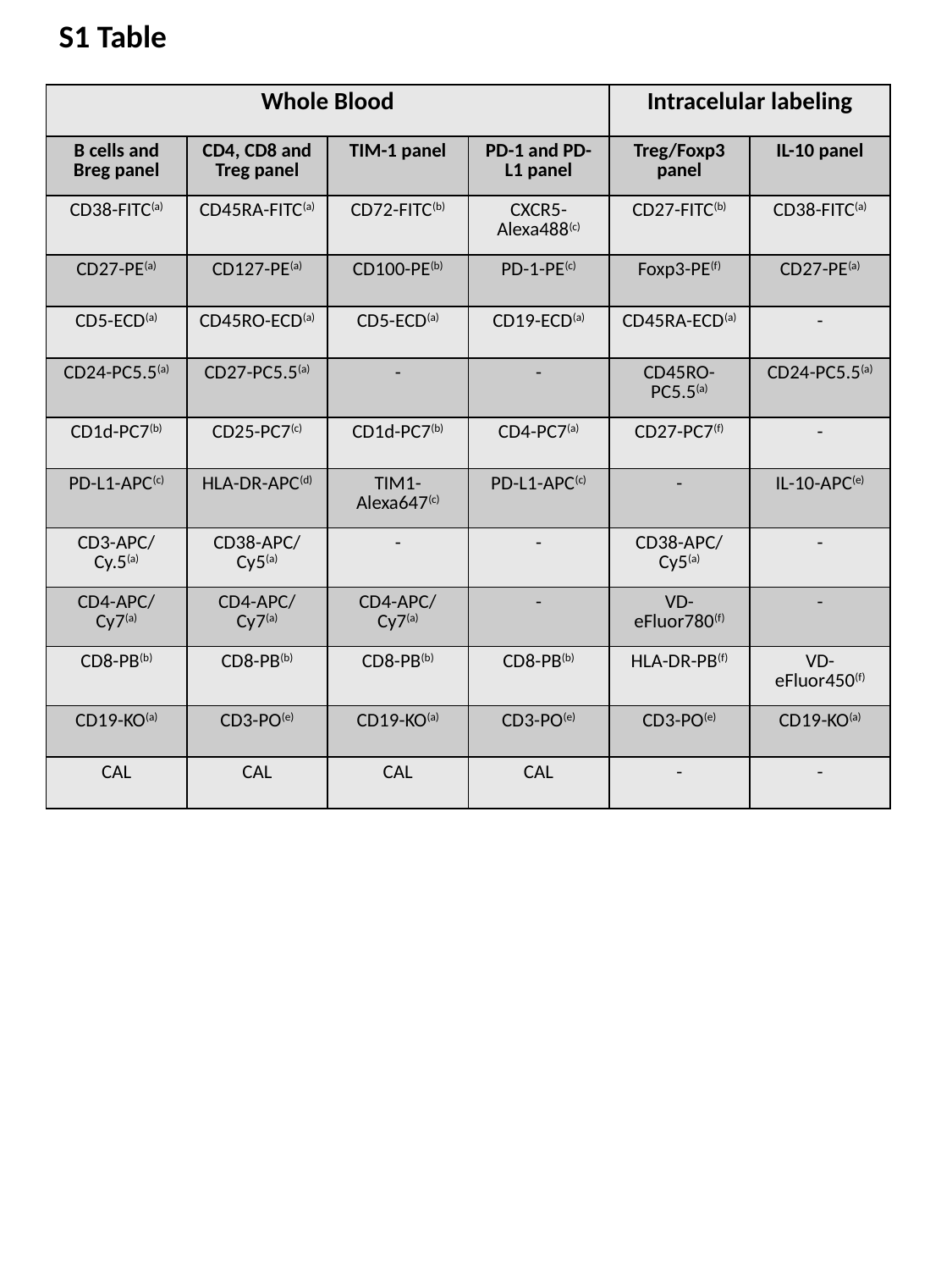

S1 Table
| Whole Blood | | | | Intracelular labeling | |
| --- | --- | --- | --- | --- | --- |
| B cells and Breg panel | CD4, CD8 and Treg panel | TIM-1 panel | PD-1 and PD-L1 panel | Treg/Foxp3 panel | IL-10 panel |
| CD38-FITC(a) | CD45RA-FITC(a) | CD72-FITC(b) | CXCR5-Alexa488(c) | CD27-FITC(b) | CD38-FITC(a) |
| CD27-PE(a) | CD127-PE(a) | CD100-PE(b) | PD-1-PE(c) | Foxp3-PE(f) | CD27-PE(a) |
| CD5-ECD(a) | CD45RO-ECD(a) | CD5-ECD(a) | CD19-ECD(a) | CD45RA-ECD(a) | - |
| CD24-PC5.5(a) | CD27-PC5.5(a) | - | - | CD45RO-PC5.5(a) | CD24-PC5.5(a) |
| CD1d-PC7(b) | CD25-PC7(c) | CD1d-PC7(b) | CD4-PC7(a) | CD27-PC7(f) | - |
| PD-L1-APC(c) | HLA-DR-APC(d) | TIM1-Alexa647(c) | PD-L1-APC(c) | - | IL-10-APC(e) |
| CD3-APC/Cy.5(a) | CD38-APC/Cy5(a) | - | - | CD38-APC/Cy5(a) | - |
| CD4-APC/Cy7(a) | CD4-APC/Cy7(a) | CD4-APC/Cy7(a) | - | VD-eFluor780(f) | - |
| CD8-PB(b) | CD8-PB(b) | CD8-PB(b) | CD8-PB(b) | HLA-DR-PB(f) | VD-eFluor450(f) |
| CD19-KO(a) | CD3-PO(e) | CD19-KO(a) | CD3-PO(e) | CD3-PO(e) | CD19-KO(a) |
| CAL | CAL | CAL | CAL | - | - |
