# Supplementary material for: Analysis of the dysregulation between regulatory B and T cells (Breg and Treg) in human immunodeficiency virus (HIV)-infected patients
Source: PLoS One. 2019 Mar 27;14(3):e0213744. doi: 10.1371/journal.pone.0213744 (PMC6436717; doi:10.1371/journal.pone.0213744)
Supplement: S2 Table — Description of the T and B-cell subsets followed in this study and the gating strategies. (PPTX) [file pone.0213744.s005.pptx]

## Slide 1
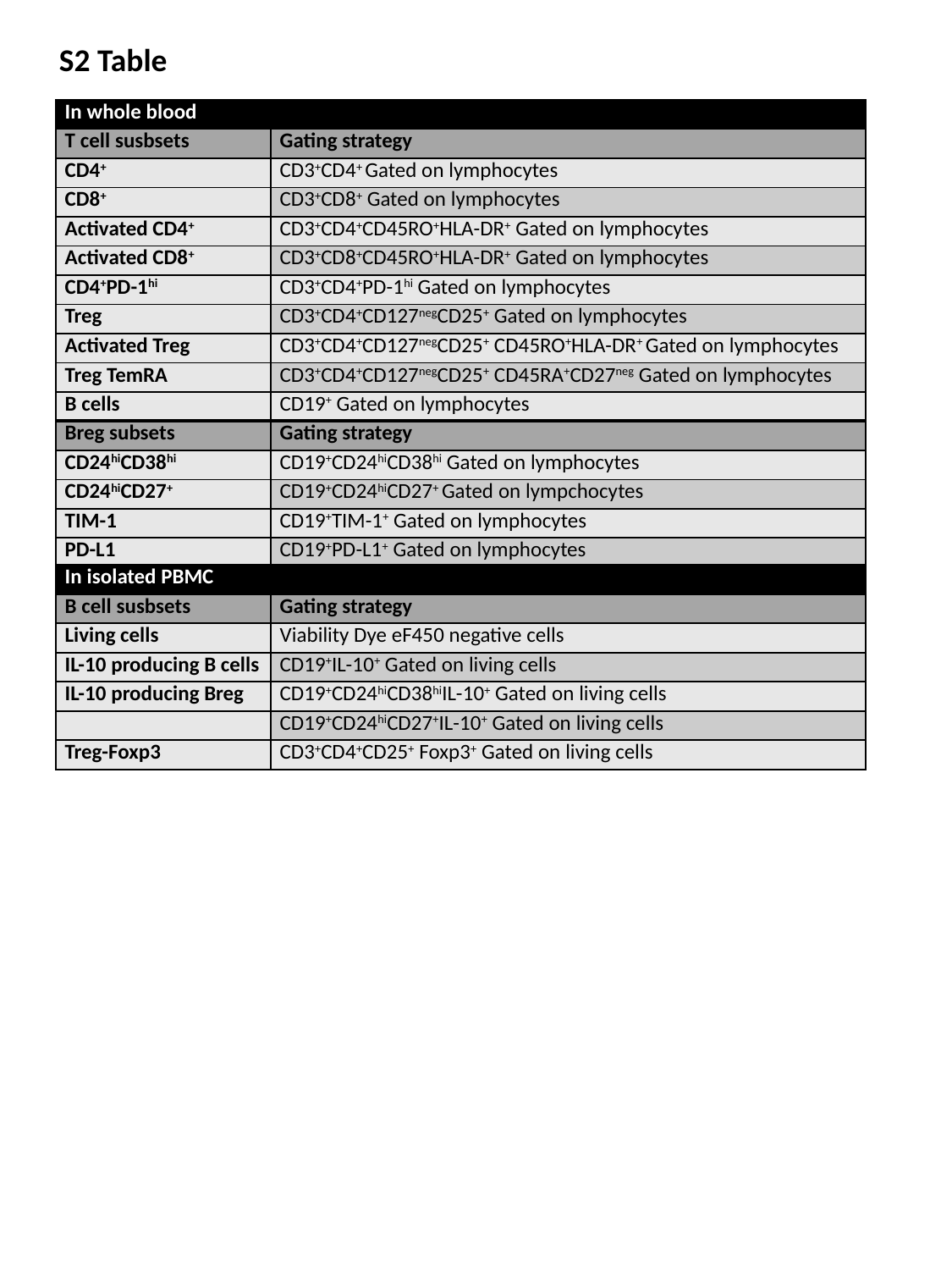

S2 Table
| In whole blood | |
| --- | --- |
| T cell susbsets | Gating strategy |
| CD4+ | CD3+CD4+ Gated on lymphocytes |
| CD8+ | CD3+CD8+ Gated on lymphocytes |
| Activated CD4+ | CD3+CD4+CD45RO+HLA-DR+ Gated on lymphocytes |
| Activated CD8+ | CD3+CD8+CD45RO+HLA-DR+ Gated on lymphocytes |
| CD4+PD-1hi | CD3+CD4+PD-1hi Gated on lymphocytes |
| Treg | CD3+CD4+CD127negCD25+ Gated on lymphocytes |
| Activated Treg | CD3+CD4+CD127negCD25+ CD45RO+HLA-DR+ Gated on lymphocytes |
| Treg TemRA | CD3+CD4+CD127negCD25+ CD45RA+CD27neg Gated on lymphocytes |
| B cells | CD19+ Gated on lymphocytes |
| Breg subsets | Gating strategy |
| CD24hiCD38hi | CD19+CD24hiCD38hi Gated on lymphocytes |
| CD24hiCD27+ | CD19+CD24hiCD27+ Gated on lympchocytes |
| TIM-1 | CD19+TIM-1+ Gated on lymphocytes |
| PD-L1 | CD19+PD-L1+ Gated on lymphocytes |
| In isolated PBMC | |
| --- | --- |
| B cell susbsets | Gating strategy |
| Living cells | Viability Dye eF450 negative cells |
| IL-10 producing B cells | CD19+IL-10+ Gated on living cells |
| IL-10 producing Breg | CD19+CD24hiCD38hiIL-10+ Gated on living cells |
| | CD19+CD24hiCD27+IL-10+ Gated on living cells |
| Treg-Foxp3 | CD3+CD4+CD25+ Foxp3+ Gated on living cells |
